# Weighted Gene Co-expression Network Analysis Reveals Different Immunity but Shared Renal Pathology Between IgA Nephropathy and Lupus Nephritis

**DOI:** 10.3389/fgene.2021.634171

**Published:** 2021-03-29

**Authors:** Ni-Ya Jia, Xing-Zi Liu, Zhao Zhang, Hong Zhang

**Affiliations:** ^1^Renal Division, Department of Medicine, Peking University First Hospital, Beijing, China; ^2^Key Laboratory of Renal Disease, Ministry of Health of China, Beijing, China; ^3^Key Laboratory of Chronic Kidney Disease Prevention and Treatment (Peking University), Ministry of Education of China, Beijing, China

**Keywords:** IgA nephropathy, lupus nephritis, weighted gene co-expression network analysis, systematic immunity, kidney immunity

## Abstract

Both IgA nephropathy (IgAN) and lupus nephritis (LN) are immunity-related diseases with a complex, polygenic, and pleiotropic genetic architecture. However, the mechanism by which the genetic variants impart immunity or renal dysfunction remains to be clarified. In this study, using gene expression datasets as a quantitative readout of peripheral blood mononuclear cell (PBMC)- and kidney-based molecular phenotypes, we analyzed the similarities and differences in the patterns of gene expression perturbations associated with the systematic and kidney immunity in IgAN and LN. Original gene expression datasets for PBMC, glomerulus, and tubule from IgAN and systemic lupus erythematosus (SLE) patients as well as corresponding controls were obtained from the Gene Expression Omnibus (GEO) database. The similarities and differences in the expression patterns were detected according to gene differential expression. Weighted gene co-expression network analysis (WGCNA) was used to cluster and screen the co-expressed gene modules. The disease correlations were then identified by cell-specific and functional enrichment analyses. By combining these results with the genotype data, we identified the differentially expressed genes causatively associated with the disease. There was a significant positive correlation with the kidney expression profile, but no significant correlation with PBMC. Three co-expression gene modules were screened by WGCNA and enrichment analysis. Among them, blue module was enriched for glomerulus and podocyte (*P* < 0.05) and positively correlated with both diseases (*P* < 0.05), mainly *via* immune regulatory pathways. Pink module and purple module were enriched for tubular epithelium and correlated with both diseases (*P* < 0.05) through predominant cell death and extracellular vesicle pathways, respectively. In genome-wide association study (GWAS) enrichment analysis, blue module was identified as the high-risk gene module that distinguishes LN from SLE and contains *PSMB8* and *PSMB9*, the susceptibility genes for IgAN. In conclusion, IgAN and LN showed different systematic immunity but similarly abnormal immunity in kidney. Immunological pathways may be involved in the glomerulopathy and cell death together with the extracellular vesicle pathway, which may be involved in the tubular injury in both diseases. Blue module may cover the causal susceptibility gene for IgAN and LN.

## Introduction

Chronic glomerulonephritis is a common cause of end-stage renal disease (ESRD), and most patients require renal replacement therapy to survive. This condition has now become a global public health problem. IgA nephropathy (IgAN) and lupus nephritis (LN) is the most common primary and secondary glomerular diseases, respectively, and tend to affect young adults, with nearly 20% of patients progressing to ESRD after 10 years because of limited drug treatment options (Mahajan et al., 2020; Natale et al., 2020). Although both diseases have complex and varied clinical manifestations, many overlaps have been noted. Specifically, both have regional and familial clustering and have hematuria and proteinuria as common clinical manifestations, with renal deposition of IgA as the pathology. The co-existence of IgAN and LN has also been reported in clinical practice (Padyukov et al., 2001; Kobak et al., 2011; Patel et al., 2019). However, the two diseases are mutually independent, with IgAN being more common in young men and LN in young women. In IgAN, IgA is the predominant immunoglobulin deposited in the kidney, while LN kidney deposition involves a “full house” pattern of immunoglobulins, including IgA. It is widely accepted that the pathogenesis of glomerulonephritis involves abnormalities in both systematic and local immunity based on the genetic background. With advances in molecular genetics, a more comprehensive understanding of the genetic variants involved in glomerulonephritis suggests that the “first hits” for glomerulonephritis are genetically determined (Couser and Johnson, 2014). In addition, due to the existence of pleiotropy, a considerable number of genes have been identified, revealing characteristics of shared genetics between IgAN and LN. Genome-wide association studies (GWAS) were used to demonstrate the existence of many susceptibility loci in both IgAN and LN as non-Mendelian-diseases. In our previous study, we revealed that IgAN and LN shared affected loci (Zhou et al., 2014), which further improved our understanding of the genetic architecture of these diseases. However, the mechanism by which these loci induce the phenotypic changes associated with the diseases remains to be elucidated. Genetic variants that interact with environmental and epigenetic risks regulate gene expression in intermediate phenotypes of peripheral blood mononuclear cell (PBMC) and kidney, which lead to clinical symptoms such as proteinuria, hematuria, renal insufficiency, and pathological changes in the kidney. These symptoms are finally manifested as different clinical syndromes, such as IgAN and LN. Therefore, in this study, we explored the systematic and regional immunity in IgAN and LN patients through weighted gene co-expression network analysis (WGCNA) of PBMC and kidney expression profiles available in public databases to improve understanding of the pathogenesis and develop targeted therapies.

## Materials and Methods

### Microarray Data

Microarray gene expression data of PBMC and kidney were collected from six studies of IgAN and systemic lupus erythematosus (SLE) from the Gene Expression Omnibus (GEO) database^1^. The PBMC data were from 12 IgAN patients and eight matched controls and from 61 SLE patients and 20 matched controls, respectively. The glomeruli data were from 27 IgAN patients and 27 controls and from 32 LN patients and 14 controls, respectively. The tubulointerstitial data were from 25 IgAN patients and 6 matched controls and from 32 LN patients and 15 matched controls, respectively (Supplementary Table 1).

### Quality Control and Normalization

**FIGURE 1:**
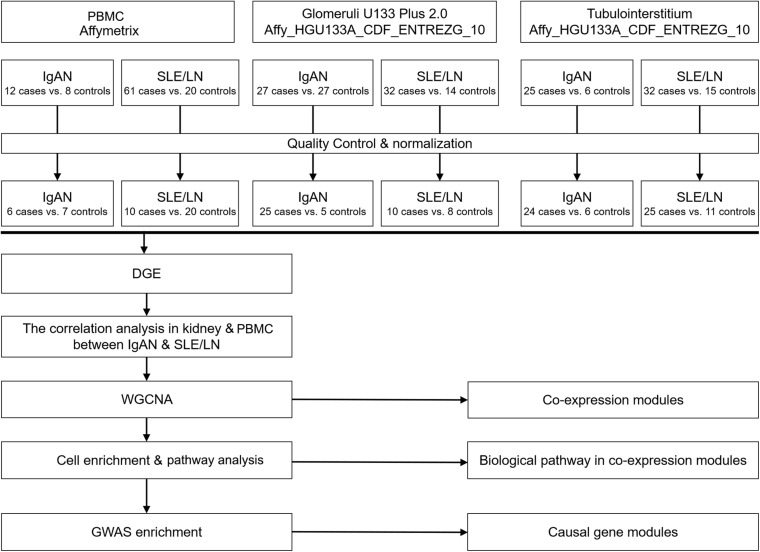
Flow chart of the study design. First, the raw data were obtained from the GEO database and subjected to quality control and standardization. Second, the correlation between different genes and disease traits was analyzed. Then, WGCNA was used to identify gene modules co-associated with IgAN and LN glomeruli or renal tubules, and co-pathogenic pathways were identified through cell-specific enrichment analysis and GO pathway enrichment analysis. Finally, the causal gene modules were identified by GWAS enrichment analysis. GEO, Gene Expression Omnibus; WGCNA, weighted gene co-expression network analysis; IgAN, IgA nephropathy; LN, lupus nephritis; GO, gene ontology; GWAS, genome-wide association study.

Individual datasets underwent stringent quality control and normalization as shown in Figure 1. Affymetrix microarrays were subjected to RMA normalization, including background correction, log2 transformation, quantile normalization, and pro summarization, using the *affy* package in R. All efforts were made to integrate available technical covariates (e.g., experimental batch, RIN). For Affymetrix microarrays, a chip scan date was used as a surrogate for the experimental batch and was extracted from the metadata. Normalized 5′/3′ bias, a measure strongly influenced by RNA degradation, was calculated for Affymetrix arrays using the AffyRNAdeg function. We balanced the case/control status across available technical covariates such that, for each study, case/control status was not significantly associated with any measured covariate (*P* > 0.05). Outliers were defined as samples with standardized sample network connectivity *Z*-scores < −2 and were removed. Batch effects were corrected with the ComBat function of the *sva* package in R. Microarray probes were re-mapped to Ensembl gene IDs (v75; February 2014 data freeze) using the *biomaRt* package in R, taking the maximum mean signal across all probes available for each gene, and using the collapseRows function (Supplementary Figures 1–6).

### Identification of Differential Gene Expression

Differential gene expression (DGE) was calculated using a linear mixed-effects model with the *nlme* package in R. Genes were then filtered to include only those that were present across all studies (13,265 Ensembl Gene IDs; listed in Supplementary Table 2). The transcriptome overlap between disease pairs was assessed using Spearman’s correlation of log2FC values across all disease pairs. Significant thresholds were determined using permutation testing to account for any study-specific factors that could potentially bias results.

### Gene Co-Expression Network Analysis

Weighted gene co-expression network analysis was used to construct a co-expression network based on normalized expression data. Using the significant correlated differential expression genes between disease pairs, individual expression datasets were combined. ComBat was used to mitigate batch effects. This normalized mega-analysis expression set was then used for all downstream network analyses.

Network analysis was performed with the *WGCNA* package using signed networks. A soft-threshold power of nine was used for all studies to achieve approximate scale-free topology (*R*^2^ > 0.8). Networks were constructed using the blockwiseModules function. The network dendrogram was created using the average linkage hierarchical clustering of the topological overlap dissimilarity matrix (1-TOM). Modules were defined as branches of the dendrogram using the hybrid dynamic tree-cutting method. Module robustness was ensured by randomly resampling (2/3 of the total) from the initial set of samples 100 times followed by consensus network analysis. Modules were defined using biweight midcorrelation (bicor), with a minimum module size of 100, a deepSplit of three, a merge threshold of 0.1, and a negative pamStage. Modules were labeled by color coding for illustration. Genes that did not fall within a specific module were assigned the color gray. We chose a minimum module size of 100, as modules with smaller sizes are more likely to capture noise. In general, we have found that, with large sample size, WGCNA is robust to changes in module parameters. Module–disease associations were evaluated using a linear mixed-effects model. We also used linear regression to test for associations between modules and several covariates or confounders (RIN, normalized 5′/3′ bias). Significance values were false discovery rate (FDR)-corrected to account for multiple comparisons. Genes within each module were prioritized based on their module membership (kME), defined as the correlation with the module eigengene.

### Cell-Specific Enrichment Analysis

Cell type-specific expression analysis of genes within each module was performed using the *pSI* package in R. Cell type-specific gene expression data were obtained from a human protein atlas of purified populations of mesangial cells, glomerular and tubule epithelial cells, and podocytes. Raw data (FPKM) were downloaded from the GEO database. Gene symbols were mapped to the Ensembl gene identifier using the *biomaRt* R package. Expression values were log2 normalized and averaged across cell type replicates. Specificity for these kidney cell types was calculated with the *specificity.index* function. Significance was assessed using Fisher’s exact test with a Psi threshold set to 0.05, followed by FDR correction of *P*-values.

### Signal Pathway Analysis

Functional enrichment of gene ontology (GO) pathways was assessed using the *gProfiler* R package with GO databases. The pathways containing between 10 and 2,000 genes were included.

### GWAS Enrichment Analysis

We used a set of GWAS summary statistics for IgAN (1,194 cases and 902 controls), SLE from European (7,219 cases and 15,991 controls) and SLE from Asian (4,492 cases and 12,675 controls) (Gharavi et al., 2011; Bentham et al., 2015; Sun et al., 2016). Gene-level analysis of the GWAS results was performed using MAGMA v1.04 to generate a gene set annotation framework that accounts for linkage disequilibrium (LD) between single nucleotide polymorphisms (SNPs) (de Leeuw et al., 2015). LD was calculated using the 1,000 Genomes European ancestry reference dataset. An annotation step was performed first, in which SNPs were mapped to genes (either hg18 or hg19 genome built, depending on the study) based on the presence of an SNP in the region between the start and stop sites of a gene. The gene-level analysis was then performed to create aggregate statistics for each gene.

To quantify the enrichment of the GWAS signal within each gene co-expression module, we calculated Spearman’s correlation coefficient between the module membership (kME) of each gene and the −log_10_ *P*-value for that gene for each GWAS. The kME is a measure between 0 and 1 of the centrality of a gene within a module; “hub genes” have kME values approaching 1, whereas genes that are not present in a module generally have kME values < 0.5. This process was performed for all module × GWAS combinations, and *P*-values were FDR corrected.

## Results

### The DGE Overlap and Expression Gradient

**FIGURE 2:**
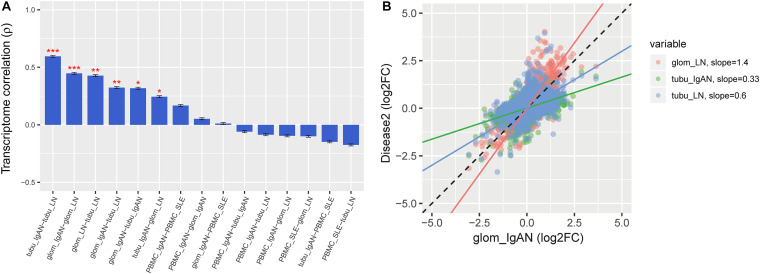
Gene expression pattern overlap across the diseases. **(A)** Rank order of the microarray transcriptome similarity between peripheral blood mononuclear cell (PBMC) and renal tissue pairs in both diseases as measured by Spearman’s correlation of differential expression (log2FC) values. **(B)** RNAseq results replicate the gradient of transcriptomic severity observed from microarray data, as measured by the regression slope, with LN-Glo > IgAN-Glo > LN-Tu > IgAN-Tu. Spearman’s ρ is shown for comparison between the microarray and region-specific RNAseq replication datasets (all *P* < 10^–14^). Data represent the mean ± SEM. *P < 0.05, **P < 0.01, and ***P < 0.001.

Comparison of DGE log2 fold change (log2FC) signatures revealed a significant overlap in renal expression profiles between IgAN and LN (*P* < 0.05), but not in PBMC (*P* > 0.05) (Figure 2A). The regression slopes for IgAN-Tu, SLE-Glo, and SLE-Tu log2FC effect sizes compared to IgAN-Glo were 0.33, 1.4, and 0.6, respectively, indicating the following gradient of transcriptomic severity: LN-glomerulus > IgAN-glomerulus > LN-tubule > IgAN-tubule (Figure 2B).

### The Co-regulated Expression Modules From WGCNA

**FIGURE 3:**
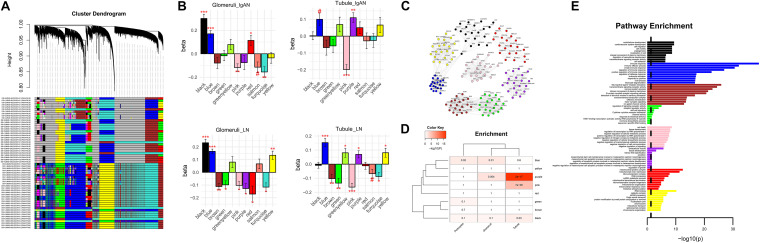
Network analysis of co-expressed genes across the diseases. **(A)** Network dendrogram of the co-expression topological overlap of genes across IgAN and LN. Colored bars show the correlation of gene expression with disease status and biological and technical covariates. As a result, 13 co-expression modules were constructed and shown in different colors. These modules ranged from large to small according to the number of genes included. **(B)** Module-level differential expression is perturbed across disease states. Plots show beta values from a linear mixed-effect model of module eigengene association with disease status [false discovery rate (FDR)-corrected ^#^*P* < 0.1, *P < 0.05, **P < 0.01, and ***P < 0.001]. **(C)** The top 20 hub genes are plotted for modules in diseases. See data Supplementary Table 2 for a complete list of module membership (kME) of the genes. Edges are weighted by the strength of the correlation between genes. **(D)** Cell-specific enrichment based on RNAseq of purified cell populations from healthy human kidney samples. Blue module was enriched for glomerulus and podocyte; both pink module and purple module were enriched for renal tubule. **(E)** Gene ontology enrichment of the top 10 pathways shown for each module among eight modules.

To obtain a more specific differential expression gene set, we performed WGCNA and identified several shared and disorder-specific co-expression modules (Figure 3A). Specifically, in both IgAN and LN, the blue module and black module were broadly upregulated in glomerulus (FDR-corrected *P* < 0.05), and the purple module was significantly upregulated in tubule (FDR-corrected *P* < 0.05). Conversely, in both diseases, the pink module was broadly downregulated in glomerulus and tubule (FDR-corrected *P* < 0.05), and the turquoise module was significantly downregulated in glomerulus (FDR-corrected *P* < 0.05) (Figure 3B). Furthermore, the top 20 hub genes and the connections for eight of the modules (blue, brown, yellow, green, red, black, pink, and purple) are shown in Figure 3C.

### Cell-Specific Enrichment Analysis to Focus Target Modules

Cell-specific analysis was performed to obtain detailed specific cytological localization of the DGE datasets. The results showed that blue module was enriched for glomerulus and podocyte (*P* = 0.009 and 0.021, respectively), and both pink module and purple module were enriched for renal tubule (*P* = 6.62 × 10^–6^ and 1.75 × 10^–17^, respectively) (Figure 3D). However, black module, which was a glomerular co-regulatory module, was not enriched in glomerular cells (*P* = 0.1) (Figure 3D), suggesting that black module was not enough to affect glomerular function. Therefore, blue, pink, and purple modules were identified as the target modules.

### Signal Pathway Behind Co-Regulated Expression Modules

Functional enrichment of GO pathways was used to characterize the biological pathways. The top 20 hub genes for the blue module (*ARPC1B*, *NMI*, *TYROBP*, *LY96*, *GBP2, VOPP1*, *PYCARD*, *CTSS*, *PIK3R2*, *PSMB9*, *HLA-DMB*, *CD53*, *S100A11*, *IFI16*, *PSMB8*, *NECAP2*, *HCLS1*, *ARHGDIB*, *HLA-DMA*, and *LAPTM5*) were intensively enriched for the immune response pathway (*P* < 0.05). The top 20 hub genes for the pink module (*MAFF*, *IER3*, *TNFRSF12A*, *HBEGF*, *KLF10*, *JUND*, *TRIB1*, *GDF15*, *ZNF165*, *MYC*, *ZFP36L2*, *ATF3*, *JUNB*, *MCL1*, *CDKN1A*, *JOSD1*, *GADD45B*, *EGR1*, *CYR61*, and *KLF6*) were significantly enriched for cell death pathway (*P* < 0.05). The top 20 genes for the purple module (*CD24P2*, *WWC1*, *TPD52L1*, *CD24P4*, *EPCAM*, *ABLIM1*, *PLS1*, *MET*, *SPINT1*, *TSPAN1*, *CDH16*, *MUC1*, *PRR5-ARHGAP8*, *F2RL1*, *BLNK*, *SCNN1A*, *PAX8*, *GPI*, *EMX2*, and *CIT*) were strongly enriched for the extracellular vesicle pathway (*P* < 0.05) (Figure 3E).

### GWAS Enrichment Analysis to Find Co-Causal Gene Module

**FIGURE 4:**
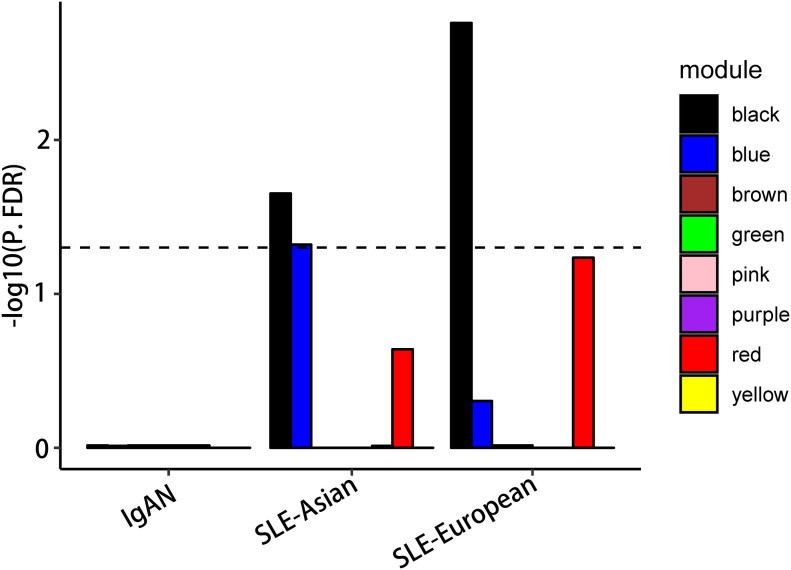
Co-correlated modules’ GWAS enrichment. Significant blue module enrichment for LN associated variants from GWAS but not for systemic lupus erythematosus (SLE); no enrichment for IgAN.

To determine how disease-associated variants affect the specific biological processes, we investigated whether any modules harbored genetic susceptibility for specific disorders using GWAS enrichment analysis. Since the SLE phenotype is high-risk in the Asian population (Sun et al., 2016), both Asian and European GWAS datasets of SLE were incorporated into our study to explore kidney-specific genes associated with severe phenotype. The result showed that the GWAS signals were significantly enriched in black module blue module in Asian-SLE (FDR-corrected *P* < 0.05), but not in Europe-SLE (FDR-corrected *P* > 0.05), suggesting blue module may represent the high-risk renal susceptibility loci (FDR-corrected *P* < 0.05). Although the black module was enriched in both European and Asian SLE populations, the above cell enrichment analysis showed that it did not produce consistent cell enrichment in its co-regulated glomerular sites (Figure 4). In addition, although the blue module was not enriched in IgAN (FDR-corrected *P* > 0.05), *PSMB8* and *PSMB9*, which were the hub genes in blue module, were identified as an IgAN susceptibility loci in an IgAN GWAS (Kiryluk et al., 2012), suggesting blue module might be associated with renal susceptibility in both IgAN and LN.

## Discussion

In this study, we investigated the common differential genes in circulating and local kidney immunity between IgAN and LN by DGE analysis using PBMC and kidney gene expression profiles. The results showed no significant overlap in differential genes between the two diseases in PBMC but showed significant overlaps in differential genes in the kidney. Subsequently, we found these genes were mainly enriched in immune regulation, cell death, and extracellular vesicle biological pathways by WGCNA and cell-specific and pathway analyses. Finally, by GWAS enrichment analysis, the blue gene module was identified to be associated with renal susceptibility in both diseases.

In this study, we found that there were not enough common differential expression genes in PBMC between IgAN and LN, suggesting the independent systematic immunity between IgAN and LN. Furthermore, the genetics of gene expression, in disease research, can be particularly illuminating when using the tissues directly impacted by the condition. Actually, the two diseases showed significant overlaps in the renal gene expression profiles. The gradient of transcriptomic severity is as follows: LN-glomerulus > IgAN-glomerulus > LN-tubule > IgAN-tubule, indicating that the DGE transcriptomic severity was stronger in LN than in IgAN, and in terms of the location of expression, the severity was more prominent in glomerulus than in tubule across the two diseases. This is consistent with the characteristics and the therapeutic effect of these diseases. LN is a manifestation of renal involvement in the multi-system damage caused by SLE, which is often combined with multi-organ damage, while the involvement of organs other than the kidney is rare in IgAN. This indicated that IgAN and LN may be the results of different effects of circulatory immunity on the same target organ (kidney). In therapy, the local renin–angiotensin system (RAS) blockers on kidney were the common foundation of the treatment of IgAN and LN (Tse et al., 2005; Pozzi, 2016). However, corticosteroids was found to be effective for the progression of LN, while it was not the first-choice recommendation for IgAN treatment (Pozzi, 2016; Almaani et al., 2017). Rituximab, which targets the systematic immune system, was showed to have significant effects on LN, but not on IgAN treatment (Sfikakis et al., 2005; Gunnarsson et al., 2007; Lafayette et al., 2017). These therapeutic similarities and differences indicate the similar kidney immunity and the different circulatory immunity between IgAN and LN. Overall, the mechanisms of the renal pathogenesis of these two diseases might be similar.

Furthermore, WGCNA and signal pathway analyses revealed that three co-regulated modules (blue module in glomerulus and pink module and purple module in tubules) were mainly enriched for immunity, cell death, and extracellular vesicle pathways, respectively, suggesting that the co-causal or co-result gene might involve in both diseases by the three biological pathways. Obviously, the immunological pathway is dominant in blue module. *PSMB8* and *PSMB9*, the hub genes in blue module encoding the immunoproteasome, were enriched for glomerulus and podocyte. Previous GWAS has identified *PSMB8* and *PSMB9* as the susceptibility genes of IgAN, and *PSMB9* was found to upregulate in the epidermis of SLE patients (Kiryluk et al., 2012; Nakamura et al., 2016). The proteasome inhibitor bortezomib was effective for the remission of LN and reducing the proteinuria in patients with IgAN (Alexander et al., 2015; Hartono et al., 2018), supporting our results that blue module is involved in the common pathogenesis of IgAN and LN *via* immunity pathways. In addition, the tubule-specific pink module was broadly downregulated in both diseases and enriched for death pathway. Regardless of the underlying etiology, the gradual progression of chronic kidney disease would lead to irreversible nephron loss (Ruiz-Ortega et al., 2020). The renal progression in IgAN correlates more closely with the severity of tubulointerstitial lesions compared with glomerular lesions (Chan et al., 2004). The death signaling pathway (TGF-β1/Smad and MAPK) and apoptosis were detected mainly in the renal tubules and interstitium in IgAN (Chan et al., 2005; Wu et al., 2009; Yao et al., 2014). Various defects in the apoptotic pathway or apoptotic cell clearance resulted in an accumulation of apoptotic debris (Pieterse and van der Vlag, 2014), which can lead to immune complex formation and the development of nephritis (Munoz et al., 2005). The mutual injury “cross-talk” network between the glomerulus and tubule (Chan et al., 2004; Wang et al., 2018) also supported the cell death pathway involved in the tubulointerstitial damage in IgAN and LN. Moreover, the purple module was enriched in the extracellular vesicle pathway, which was involved in the tubular injury of IgAN and LN. It has been reported that the marked increase in the number of urinary exosomes produced by renal tubular epithelial cells (Khurana et al., 2017) in IgAN was correlated with higher levels of proteinuria, leading to exacerbated tubular injury and greater histologic activity (Feng et al., 2018). Furthermore, urinary exosomal miR-135b-5p, miR-107, and miR-31 produced mainly by renal tubular cells were correlated with the prognosis of LN (Garcia-Vives et al., 2020). In accordance with the upregulated hub genes identified in our study, *EPCAM* and *MUC1* were strongly expressed on the renal tubular epithelium and were assumed to contribute to tubulointerstitial lesion formation (Staubach et al., 2018; Hagiyama et al., 2019). All evidence support that the upregulated DGEs in purple module might play a transport role *via* the extracellular vesicle pathway and participate in the pathological changes in the renal tubules in IgAN and LN.

The GWAS enrichment analysis was taken out in both IgAN and SLE. It is important that not only the European SLE population but also the Asians with usually high-risk susceptibility were included. The results finally showed that the glomeruli-related blue module was enriched in SLE-Asian GWAS rather than SLE-European, suggesting that the blue module was the high-risk and nephrospecific susceptibility gene module in LN. In addition, *PSMB8* and *PSMB9*, the hub genes in blue module, have been identified as the susceptibility genes for IgAN by GWAS (Kiryluk et al., 2012), although our GWAS enrichment analysis suggested blue module was not enriched in IgAN, which might be limited by the samples size of IgAN data. More importantly, *PSMB8* and *PSMB9* were found to be involved in the transformation of peripheral blood proteasome to immunoproteasome, which is closely related to the exacerbation of IgAN (Peruzzi et al., 2020). Furthermore, *PSMB8* and *PSMB9* have been identified as the core genes related to immune cells in kidney and a “cross-talk” gene of glomerulus and tubules by two other LN expression profile studies (Cao et al., 2019; Yao et al., 2020). These shreds of evidence suggested that blue module may cover the co-causal kidney gene for IgAN and LN. Here, we also noted that the black module was enriched in both Asian and European GWAS of SLE, in line with its unenrichment of glomerular cells, indicating that the black module was the systematic susceptible gene module of SLE rather than nephrospecific. Consistently, no hub gene in black module was identified as the susceptibility gene for IgAN in currently published GWAS (Feehally et al., 2010; Gharavi et al., 2011; Yu et al., 2011; Kiryluk et al., 2014; Li et al., 2015, 2020), supporting that black module was not the common kidney susceptibility gene module of IgAN and LN.

Nowadays, there is no report about the correlation between IgAN and LN expression profiles, and our study first demonstrated that IgAN and LN have independent systematic immunity, but share common susceptibility genes in kidney, and identified the shared causal gene may act dominantly *via* immunological pathways to trigger glomerular injury, while the death and extracellular vesicle pathways were implicated as the common mechanism underlying the development of renal tubular lesions. These findings expand our understanding of the pathology of IgAN and LN and provide a framework for future investigations into the mechanisms underlying transcriptomic alterations. The expression profiles analysis across two diseases provided a method of comprehensive investigation between diseases. The use of omics database avoided the additional economic and time investment brought by sample testing. Furthermore, the WGCNA was used greatly and simplified the workload base on the clustering idea. More importantly, more credible information was obtained in this study by integrating transcriptome and genomics, highlighting potential biomarkers and common therapeutic targets. In addition, some limitations of our research should be noted. First, due to the lack of specific clinical information for the data downloaded from the GEO, it was not possible to clarify the relationship between samples and clinical features to provide further information. Furthermore, the identification of the co-biological pathways and co-susceptibility gene module was mainly derived from the public datasets, and more reliable experiments and replications in independent populations are needed in the future.

## Conclusion and Future Research

Using a bioinformatics algorithm, we demonstrated that IgAN and LN have independent systematic immunity, but share common susceptibility genes in the kidney. We found that cell–cell signaling events rooted in genetic risk were involved in both diseases. The co-causal gene may act dominantly *via* immunological pathways to trigger glomerular injury, while the death and extracellular vesicle pathways were implicated as the common mechanism underlying the development of renal tubular lesions. We also identified hub genes that were jointly involved in both diseases. These findings expand our understanding of the pathology of IgAN and LN and provide a framework for future investigations into the mechanisms underlying transcriptomic alterations at the gene-specific and isoform levels. Moreover, the information obtained in this study highlights potential biomarkers and common therapeutic targets, especially those shared by the two diseases.

## Data Availability Statement

The original contributions presented in the study are included in the article/Supplementary Material, further inquiries can be directed to the corresponding author/s.

## Author Contributions

N-YJ and HZ conceived the study, generated the original hypothesis, and designed the research topic. N-YJ and X-ZL performed the research, conducted the data analysis, and drafted the manuscript. N-YJ, X-ZL, ZZ, and HZ checked the data and analyzed the results. HZ provided financial support. All authors reviewed/edited the manuscript and approved the final version.

## Conflict of Interest

The authors declare that the research was conducted in the absence of any commercial or financial relationships that could be construed as a potential conflict of interest.

## Footnotes

1 http://www.ncbi.nlm.nih.gov/geo/

## Supplementary Material

Supplementary Figure 1 Quality control of expression data from PBMC samples in IgAN.

Supplementary Figure 2 Quality control of expression data from PBMC samples in SLE.

Supplementary Figure 3 Quality control of expression data from glomerular samples in IgAN.

Supplementary Figure 4 Quality control of expression data from glomerular samples in LN.

Supplementary Figure 5 Quality control of expression data from renal tubular samples in IgAN.

Supplementary Figure 6 Quality control of expression data from renal tubular samples in LN. From Supplementary Figure 1 to Supplementary Figure 6, for each microarray dataset, we show several quality control plots, including expression boxplots and histograms. Outlier detection was determined based on standardized network connectivity *Z*-scores. Multidimensional scaling (MDS) plots show sample clustering by the first two expression principal components. Groups were balanced by available covariates and potential confounding factors.

Supplementary Table 1 Gene expression microarray datasets included in this study.

Supplementary Table 2 Differential gene expression summary statistics from microarray meta-analysis and RNAseq replication datasets.
