# Supplementary figures and images for: Weighted Gene Co-expression Network Analysis Reveals Different Immunity but Shared Renal Pathology Between IgA Nephropathy and Lupus Nephritis
Source: Front Genet. 2021 Mar 29;12:634171. doi: 10.3389/fgene.2021.634171 (PMC8039522; doi:10.3389/fgene.2021.634171)

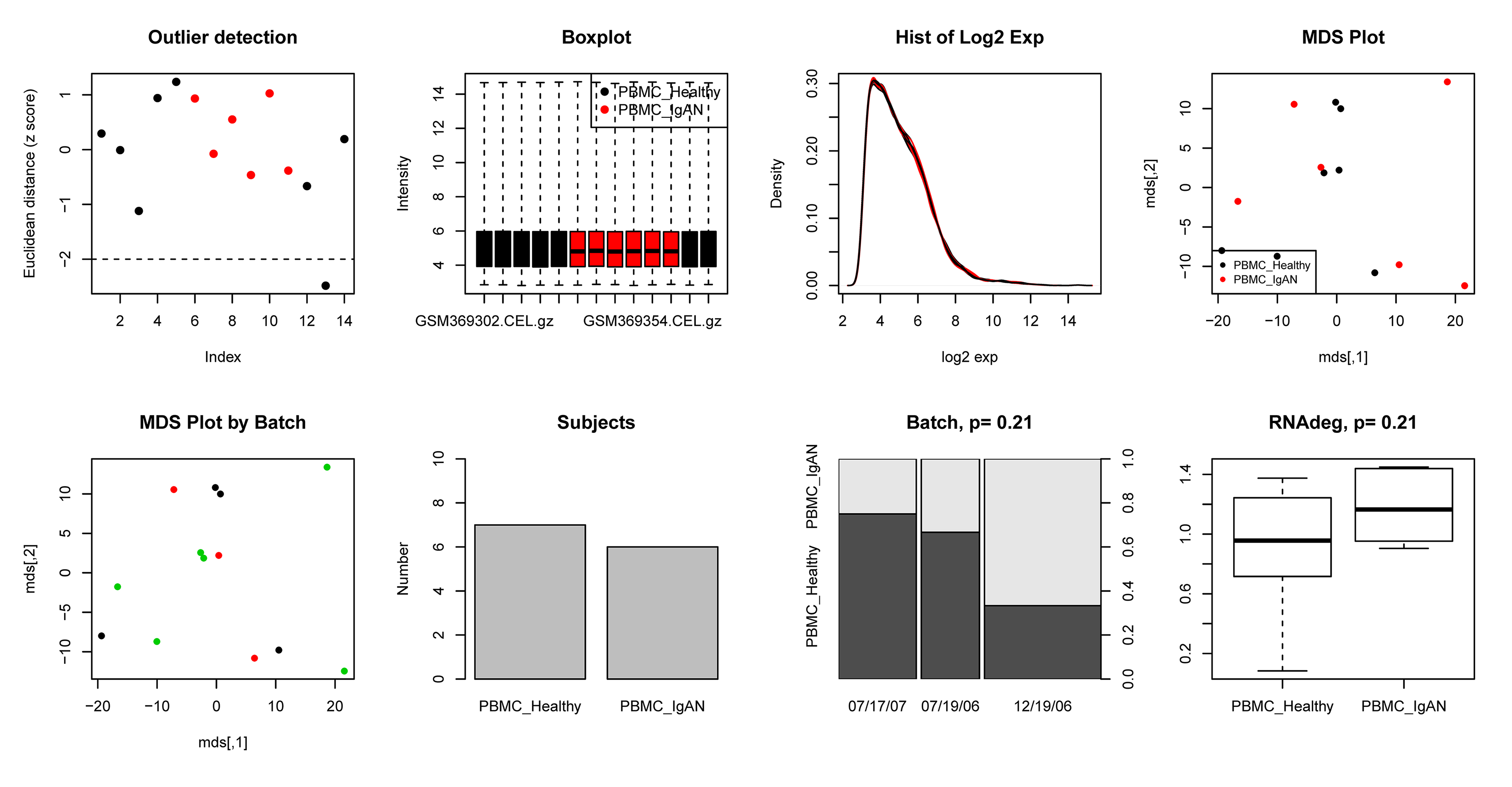

Supplement: Supplementary Figure 1 — Quality control of expression data from PBMC samples in IgAN. [file Image_1.TIF]

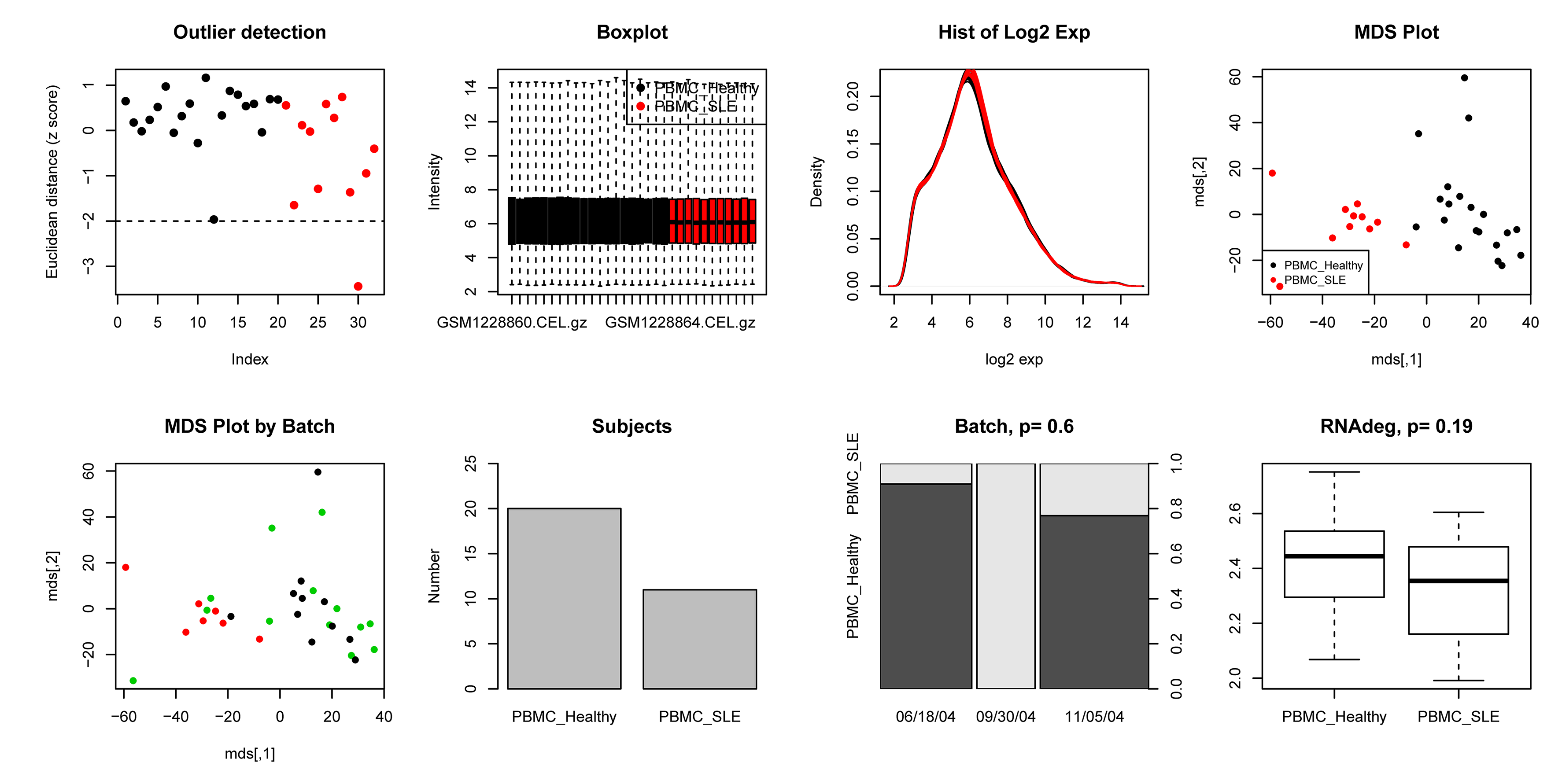

Supplement: Supplementary Figure 2 — Quality control of expression data from PBMC samples in SLE. [file Image_2.TIF]

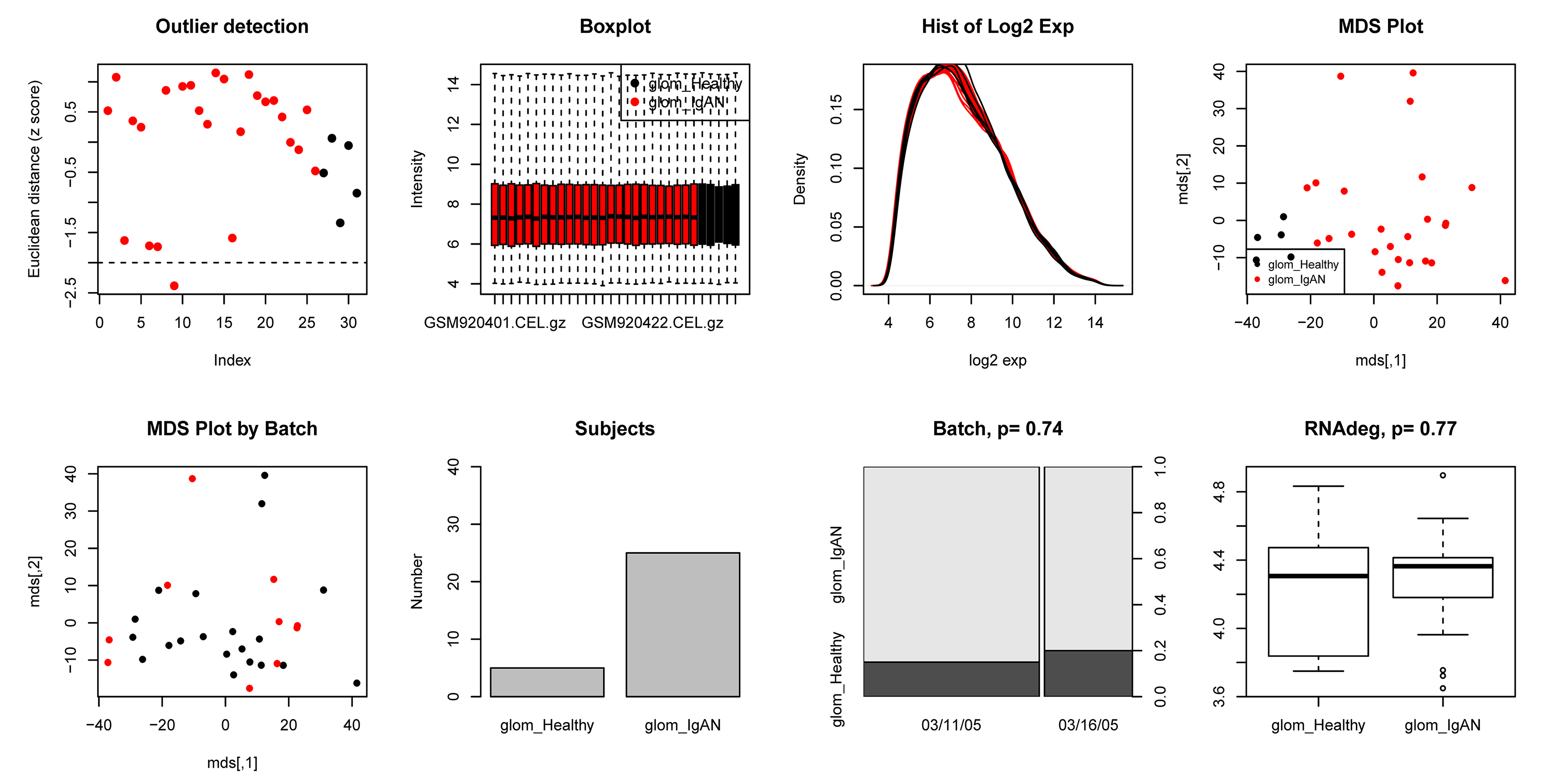

Supplement: Supplementary Figure 3 — Quality control of expression data from glomerular samples in IgAN. [file Image_3.TIF]

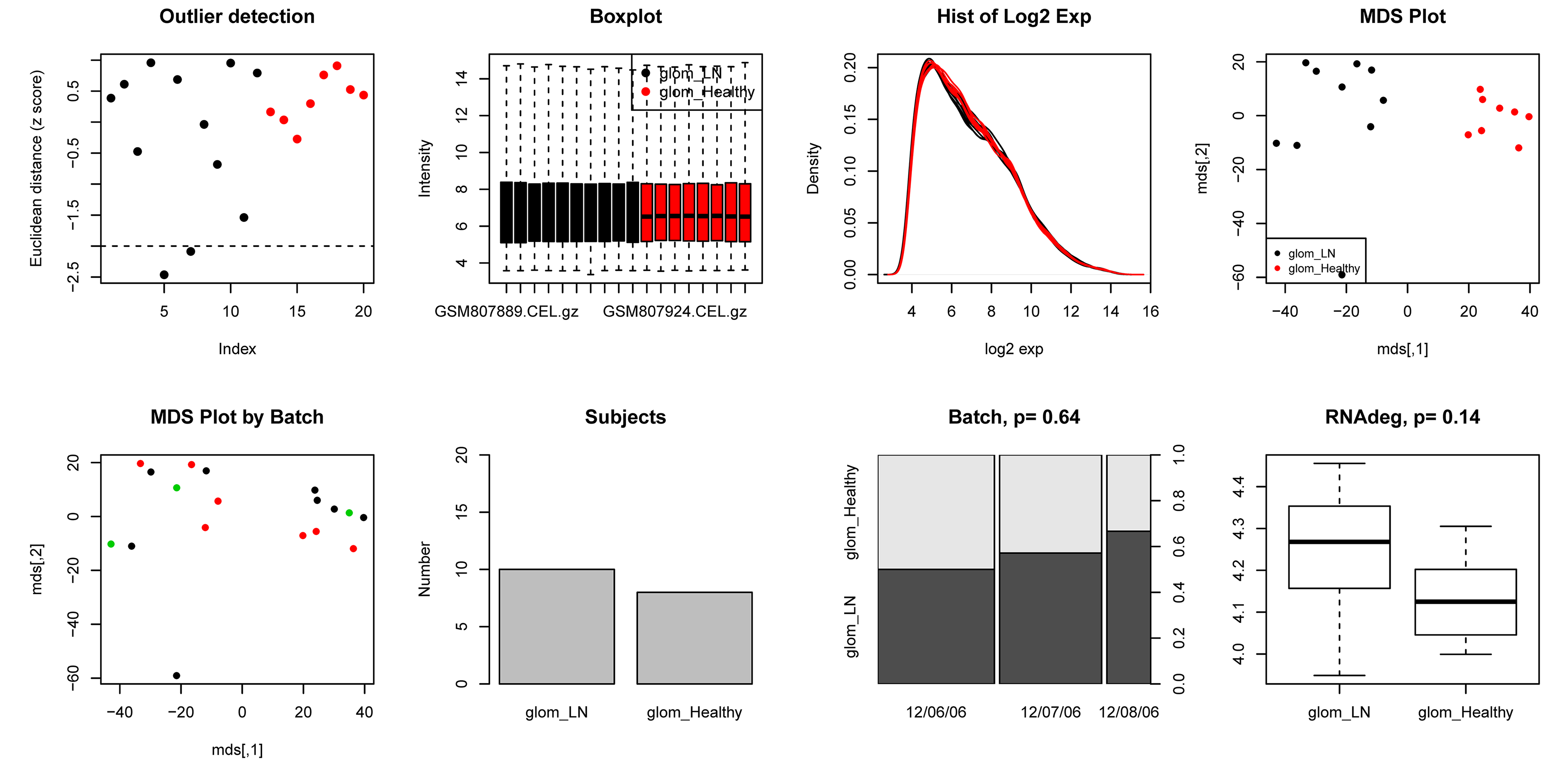

Supplement: Supplementary Figure 4 — Quality control of expression data from glomerular samples in LN. [file Image_4.TIF]

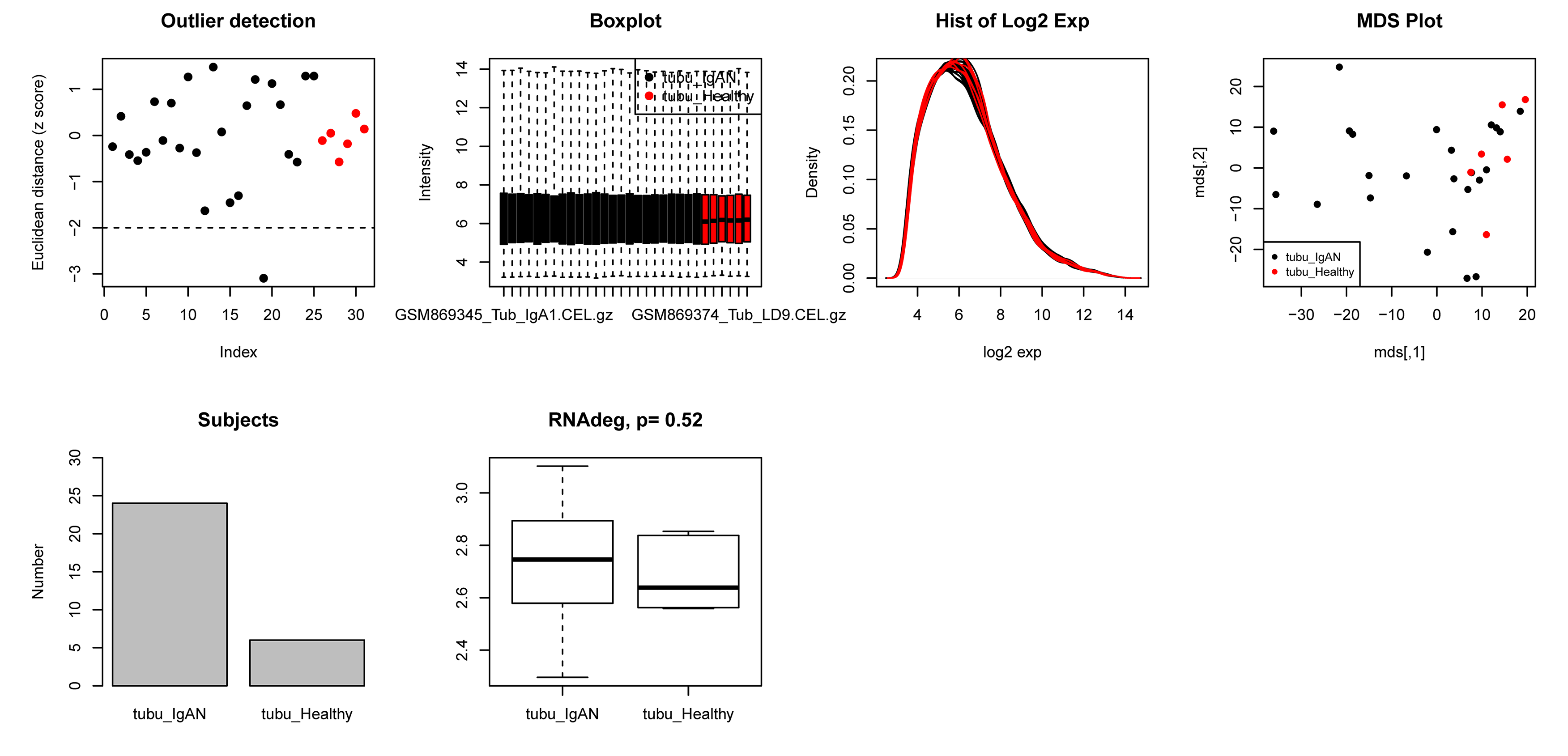

Supplement: Supplementary Figure 5 — Quality control of expression data from renal tubular samples in IgAN. [file Image_5.TIF]

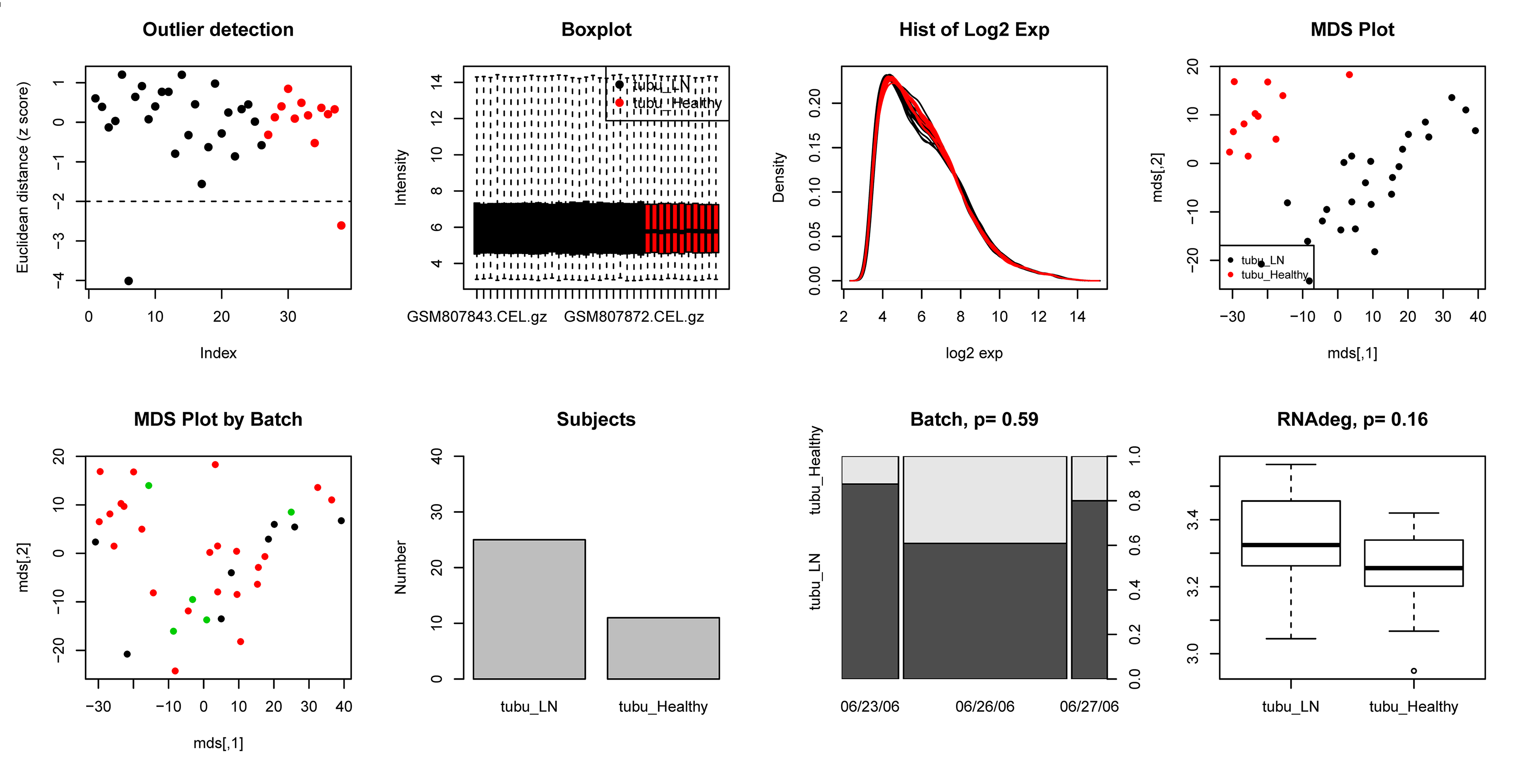

Supplement: Supplementary Figure 6 — Quality control of expression data from renal tubular samples in LN. From Supplementary Figure 1 to Supplementary Figure 6, for each microarray dataset, we show several quality control plots, including expression boxplots and histograms. Outlier detection was determined based on standardized network connectivity Z-scores. Multidimensional scaling (MDS) plots show sample clustering by the first two expression principal components. Groups were balanced by available covariates and potential confounding factors. [file Image_6.TIF]
