# Supplementary material for: Weighted Gene Co-expression Network Analysis Reveals Different Immunity but Shared Renal Pathology Between IgA Nephropathy and Lupus Nephritis
Source: Front Genet. 2021 Mar 29;12:634171. doi: 10.3389/fgene.2021.634171 (PMC8039522; doi:10.3389/fgene.2021.634171)
Supplement: Supplementary Table 1 — Gene expression microarray datasets included in this study. [file Table_1.DOCX]

**Table S1 Gene expression microarray datasets included in this study**

| **Disease** | **Tissue** | **GEO** | **Platform** | **Case** | **Control** | **Case**  **(QC)** | **Control**  **(QC)** |
| --- | --- | --- | --- | --- | --- | --- | --- |
| **IgAN** | Whole blood | GSE14795 | Affymetrix | 12 | 8 | 6 | 7 |
| **IgAN** | Glomeruli | GSE37460 | Affy_HGU133A_CDF_ENTREZG_10] | 27 | 27 | 25 | 5 |
| **IgAN** | Tubulointerstitium | GSE35488 | Affy_HGU133A_CDF_ENTREZG_10 | 25 | 6 | 24 | 6 |
| **SLE** | PBMCs | GSE50772 | Affymetrix | 61 | 20 | 10 | 20 |
| **SLE** | Glomeruli | GSE32591 | Affy_HGU133A_CDF_ENTREZG_10 | 32 | 14 | 10 | 8 |
| **SLE** | Tubulointerstitium | GSE32591 | Affy_HGU133A_CDF_ENTREZG_10 | 32 | 15 | 25 | 11 |

**Abbreviations:** IgAN, IgA nephropathy; SLE, systemic lupus erythematosus; PBMCs, peripheral blood mononuclear cells; GEO, gene expression omnibus; QC, quality control.
